# Efficacy and Renal Safety of Sacubitril/Valsartan in Heart Failure Patients With Chronic Kidney Disease: A Prospective Observational Study

**DOI:** 10.7759/cureus.83141

**Published:** 2025-04-28

**Authors:** Anum Amjad, Arva Zahid, Nayab Sami, Qaiser Wadud, Mahda Omer, Muhammad Abbas Awan, Irshad Muhammad

**Affiliations:** 1 Medicine, University Hospitals Birmingham, Birmingham, GBR; 2 Medicine, Shalamar Hospital, Lahore, PAK; 3 Cardiology, Shaikh Zayed Hospital, Lahore, PAK; 4 Cardiology, Birmingham Heartlands Hospital, Birmingham, GBR; 5 General and Internal Medicine, Khyber Teaching Hospital, Peshawar, PAK; 6 Internal Medicine, Ahfad University for Women, Omdurman, SDN; 7 General and Internal Medicine, POF (Pakistan Ordnance Factories) Hospital, Wah Cantt, Rawalpindi, PAK; 8 General and Internal Medicine, University Hospitals Birmingham NHS Foundation Trust, Birmingham, GBR; 9 Medicine, Khyber Teaching Hospital, Peshawar, PAK

**Keywords:** chronic kidney disease, entresto, heart failure, hospitalization, left ventricular ejection fraction, renal function, sacubitril/valsartan

## Abstract

Background: Patients with heart failure (HF) and chronic kidney disease (CKD) often face therapeutic challenges, necessitating optimized treatment strategies to improve clinical outcomes. This study aims to evaluate the effectiveness of sacubitril/valsartan (Entresto) in improving clinical outcomes, reducing hospitalizations, and preserving renal function in HF patients with CKD, with a primary focus on changes in renal function (estimated glomerular filtration rate (eGFR)), and secondary outcomes, including heart performance (left ventricular ejection fraction (LVEF)) and hospitalization rates.

Methodology: This prospective observational study was conducted from January 2023 to December 2023. A total of 196 consecutively enrolled HF patients with CKD stages 2-4 were included and administered sacubitril/valsartan at FDA-recommended doses. Assessments of eGFR, hospitalization rates, LVEF, and adverse events were performed at three, six, and 12 months. Statistical analysis was conducted using SPSS version 25 (IBM Corp., Armonk, NY), and a p-value < 0.05 was considered significant.

Results: The mean eGFR improved significantly from 45.8 ± 12.4 mL/min/1.73 m² to 48.5 ± 11.5 mL/min/1.73 m² after 12 months (p = 0.032), representing a 5.90% increase. Hospitalizations decreased markedly from 210 to 48 events, with hospitalization rates dropping from 65.31% to 17.35%. LVEF improved significantly from 32.5 ± 6.7% to 41.5 ± 6.1% (27.69% increase). The New York Heart Association (NYHA) functional class also improved: class II patients increased from 43.88% to 63.27%, while class IV patients declined from 16.33% to 9.18%. Adverse events included hyperkalemia in 11.22%, symptomatic hypotension in 8.16%, and worsening renal function in 5.10% of patients; 7.14% of patients discontinued therapy due to side effects.

Conclusion: Sacubitril/valsartan demonstrated significant improvement in cardiac function, reduction in hospitalization rates, and preservation of renal function in HF patients with CKD, supporting its clinical utility in this population.

## Introduction

Heart failure (HF) remains a major global public health concern, affecting millions and exerting a significant burden on healthcare systems worldwide [1]. A considerable proportion of patients with HF also suffer from chronic kidney disease (CKD), complicating disease management and increasing morbidity and mortality [2]. The cardiorenal syndrome (CRS) describes the bidirectional relationship between HF and CKD, where dysfunction of one organ leads to deterioration of the other; this is clinically classified into five types, with CRS type 2 representing chronic HF leading to progressive CKD [3].

Traditional HF therapies, such as angiotensin-converting enzyme inhibitors (ACEIs) and angiotensin receptor blockers (ARBs), have demonstrated cardiovascular benefits but are associated with potential renal risks, making their use challenging in patients with CKD [4,5]. Sacubitril/valsartan, a novel angiotensin receptor-neprilysin inhibitor (ARNI), offers a promising alternative. It has been shown to be superior to enalapril in reducing cardiovascular mortality and HF hospitalizations [6]. Sacubitril/valsartan simultaneously inhibits the renin-angiotensin-aldosterone system (RAAS) and enhances natriuretic peptide activity [7].

This dual mechanism is particularly beneficial in HF, improving myocardial function, mitigating ventricular remodeling, and enhancing diuresis. Improved volume overload control is especially advantageous in patients with both HF and CKD [8]. Although sacubitril/valsartan demonstrates efficacy in HF, its effects on renal function require cautious interpretation. Metabolic alterations in CKD can influence drug pharmacokinetics, increasing risks such as hyperkalemia and worsening renal function [9,10].

The clinical trials and evidence suggest that sacubitril/valsartan may offer renal protection in addition to cardiac benefits [11]. Recent guidelines emphasize careful dose titration rather than avoidance in patients with CKD receiving sacubitril/valsartan. Further research is needed to assess its long-term efficacy, optimal dosing, and safety across different stages of renal impairment. This study aims to evaluate the effectiveness of sacubitril/valsartan (Entresto) in improving clinical outcomes, reducing hospitalizations, and preserving renal function in patients with HF and CKD. The primary objective was to assess the impact of sacubitril/valsartan on renal function, as measured by changes in estimated glomerular filtration rate (eGFR), in HF patients with CKD. The secondary objectives were to evaluate the effects of sacubitril/valsartan on heart performance, measured by changes in left ventricular ejection fraction (LVEF), and to investigate its influence on hospitalization rates in this patient population.

## Materials and methods

Study design and setting

This was a prospective observational study conducted at the Khyber Teaching Hospital, Peshawar, over a period of one year, from January 2023 to December 2023.

Inclusion and exclusion criteria

The study focused on adults (≥18 years) diagnosed with heart failure with reduced ejection fraction (HFrEF) and CKD stages 2-4 (eGFR = 15-89 mL/min/1.73 m²) who were prescribed sacubitril/valsartan as part of their treatment for HF. Patients with missing data, CKD stage 5 or those on dialysis, a history of angioedema or intolerance to sacubitril/valsartan, severe liver failure (Child-Pugh C), or who were pregnant or breastfeeding were excluded.

Sample size and sampling technique

A total of 196 patients were consecutively enrolled using a convenience sampling method, based on the availability of eligible patients during the one-year study period. Given the observational nature of the study, no formal sample size calculation was performed. Instead, the sample size was determined pragmatically by aiming to include as many eligible participants as possible to enhance the study’s external validity and to capture a representative patient population. Consecutive enrollment helped mitigate potential selection bias inherent in convenience sampling.

Dosage according to FDA guidelines

Sacubitril/valsartan was administered twice daily in doses of 24/26 mg, 49/51 mg, or 97/103 mg, depending on prior ACEI/ARB use, baseline renal function, and blood pressure. Dose escalation was attempted every two to four weeks with the aim of reaching the target dose of 97/103 mg twice daily, contingent upon patient tolerability. Up-titration success rates and reasons for failure (e.g., hypotension, hyperkalemia, and intolerance) were tracked and recorded.

Data collection

Baseline data, including demographics, comorbidities, initial eGFR, N-terminal pro-B-type natriuretic peptide (NT-proBNP) levels, and medication history, were collected. Patients were evaluated at three, six, and 12 months to assess changes in renal function (eGFR trajectory), hospitalization rates, LVEF, and adverse events (e.g., hyperkalemia, symptomatic hypotension, and worsening renal function).

Analysis of hospitalization rates

Hospitalization rates were tracked and analyzed as a primary outcome for patient outcomes. The number of hospitalizations due to HF exacerbations was recorded at baseline and at three, six, and 12 months of follow-up. For each patient, the total number of hospital admissions related to HF during the study period was counted. The length of stay for each hospitalization was noted, and the rate of hospitalizations per patient over the study duration was calculated. The percentage of patients hospitalized at each follow-up time point (three, six, and 12 months) was also computed and compared across different study time points to assess changes in hospitalization rates. Trends in hospitalization rates were evaluated using statistical analysis to determine if sacubitril/valsartan significantly reduced hospitalizations due to HF.

Statistical analysis

Data analysis was conducted using SPSS version 25 (IBM Corp., Armonk, NY). Continuous variables were assessed for normality and presented as mean ± standard deviation for normally distributed variables or median (interquartile range) for non-normally distributed variables. Categorical variables were expressed as frequencies and percentages. Chi-square tests were used to assess associations between categorical variables, and p-values <0.05 were considered statistically significant.

Ethical approval

The study was approved by the Institutional Review Board of Khyber Medical College, Peshawar (Approval Number: 952/DGIM/KMC). Informed consent was obtained from all participants before enrollment.

## Results

The study included 196 patients with a mean age of 65.4 ± 10.2 years. Among them, 124 (63.27%) were men and 72 (36.73%) were women. The most common comorbidities were hypertension, present in 132 patients (67.35%), and diabetes mellitus, present in 96 patients (48.98%). In terms of HF severity, 86 patients (43.88%) were classified as New York Heart Association (NYHA) class II, 78 (39.80%) as class III, and 32 (16.33%) as class IV. The mean baseline eGFR was 45.8 ± 12.4 mL/min/1.73 m², while NT-proBNP levels averaged 3400 ± 850 pg/mL. The baseline LVEF was 32.5 ± 6.7% (Table 1).

**Table 1:** Baseline characteristics and outcomes of HF patients with CKD receiving sacubitril/valsartan. HF: heart failure; CKD: chronic kidney disease; eGFR: estimated glomerular filtration rate; LVEF: left ventricular ejection fraction.

Category	Characteristic	Baseline (n = 196)	3 months (n = 196)	6 months (n = 196)	12 months (n = 196)
Demographics	Age (mean ± SD, years)	65.4 ± 10.2	-	-	-
	Male	124 (63.27%)	-	-	-
	Female	72 (36.73%)	-	-	-
Comorbidities	Hypertension	132 (67.35%)	-	-	-
	Diabetes mellitus	96 (48.98%)	-	-	-
Heart failure severity	NYHA Class II	86 (43.88%)	-	-	-
	NYHA Class III	78 (39.80%)	-	-	-
	NYHA Class IV	32 (16.33%)	-	-	-
Renal function (eGFR)	Baseline eGFR (mL/min/1.73 m²)	45.8 ± 12.4	46.3 ± 12.0 (+1.09%)	47.1 ± 11.8 (+2.84%)	48.5 ± 11.5 (+5.90%)
Cardiac function (LVEF)	Baseline LVEF (%)	32.5 ± 6.7	35.2 ± 6.5 (+8.31%)	38.4 ± 6.3 (+18.15%)	41.5 ± 6.1 (+27.69%)
Hospitalization	Patients hospitalized (%)	128 (65.31%)	74 (37.76%)	52 (26.53%)	34 (17.35%)
	Total hospital admissions	210	98	70	48
Adverse events	Hyperkalemia	0 (0%)	4 (2.04%)	10 (5.10%)	22 (11.22%)
	Symptomatic hypotension	0 (0%)	5 (2.55%)	10 (5.10%)	16 (8.16%)
	Worsening renal function	0 (0%)	0 (0%)	5 (2.55%)	10 (5.10%)
	Angioedema	0 (0%)	0 (0%)	0 (0%)	2 (1.02%)

Over the 12-month follow-up, renal function showed a consistent upward trend (Table 2). The mean eGFR improved from 45.8 ± 12.4 mL/min/1.73 m² at baseline to 46.3 ± 12.0 (an increase of +1.09%) at three months, 47.1 ± 11.8 (+2.84%) at six months, and 48.5 ± 11.5 (+5.90%) at 12 months, suggesting a gradual preservation of kidney function among the patients receiving sacubitril/valsartan. The changes at six months and 12 months are statistically significant, with p-values of p = 0.03 and p < 0.001, respectively, whereas the change at three months was not statistically significant (p = 0.15).

**Table 2:** Change in renal function (eGFR) over 12 months. * P-value < 0.05 is significant. eGFR: estimated glomerular filtration rate.

Timepoint	Mean eGFR (mL/min/1.73 m²)	Change from baseline (%)	p-value
Baseline	45.8 ± 12.4	-	-
3 months	46.3 ± 12.0	1.09%	0.15
6 months	47.1 ± 11.8	2.84%	0.03*
12 months	48.5 ± 11.5	5.90%	<0.001*

Table 3 shows that the number of hospitalizations significantly declined over the course of treatment. At baseline, 128 patients (65.31%) had been hospitalized, accounting for 210 total hospital admissions. By three months, this proportion dropped to 74 patients (37.76%) with 98 admissions, followed by 52 patients (26.53%) with 70 admissions at six months, and 34 patients (17.35%) with 48 admissions by 12 months. All changes in LVEF at three months, six months, and 12 months are statistically significant with p < 0.001. These results indicate a marked reduction in hospitalization frequency with sacubitril/valsartan therapy.

**Table 3:** Reduction in hospitalization rates.

Timepoint	Number of patients hospitalized (%)	Total hospitalizations
Baseline (prior year)	128 (65.31%)	210
3 months	74 (37.76%)	98
6 months	52 (26.53%)	70
12 months	34 (17.35%)	48

Cardiac function improved significantly during the study, as shown in Table 4. The mean LVEF increased from 32.5 ± 6.7% at baseline to 35.2 ± 6.5% at three months (+8.31%), 38.4 ± 6.3% at six months (+18.15%), and 41.5 ± 6.1% at 12 months (+27.69%), reflecting substantial recovery in left ventricular performance under sacubitril/valsartan therapy.

**Table 4:** Improvement in left ventricular ejection fraction (LVEF). * P-value < 0.05 is significant.

Timepoint	Mean LVEF (%) ± SD	Change from baseline (%)	p-value
Baseline	32.5 ± 6.7	-	-
3 months	35.2 ± 6.5	8.31%	<0.001*
6 months	38.4 ± 6.3	18.15%	<0.001*
12 months	41.5 ± 6.1	27.69%	<0.001*

**Figure 1:**
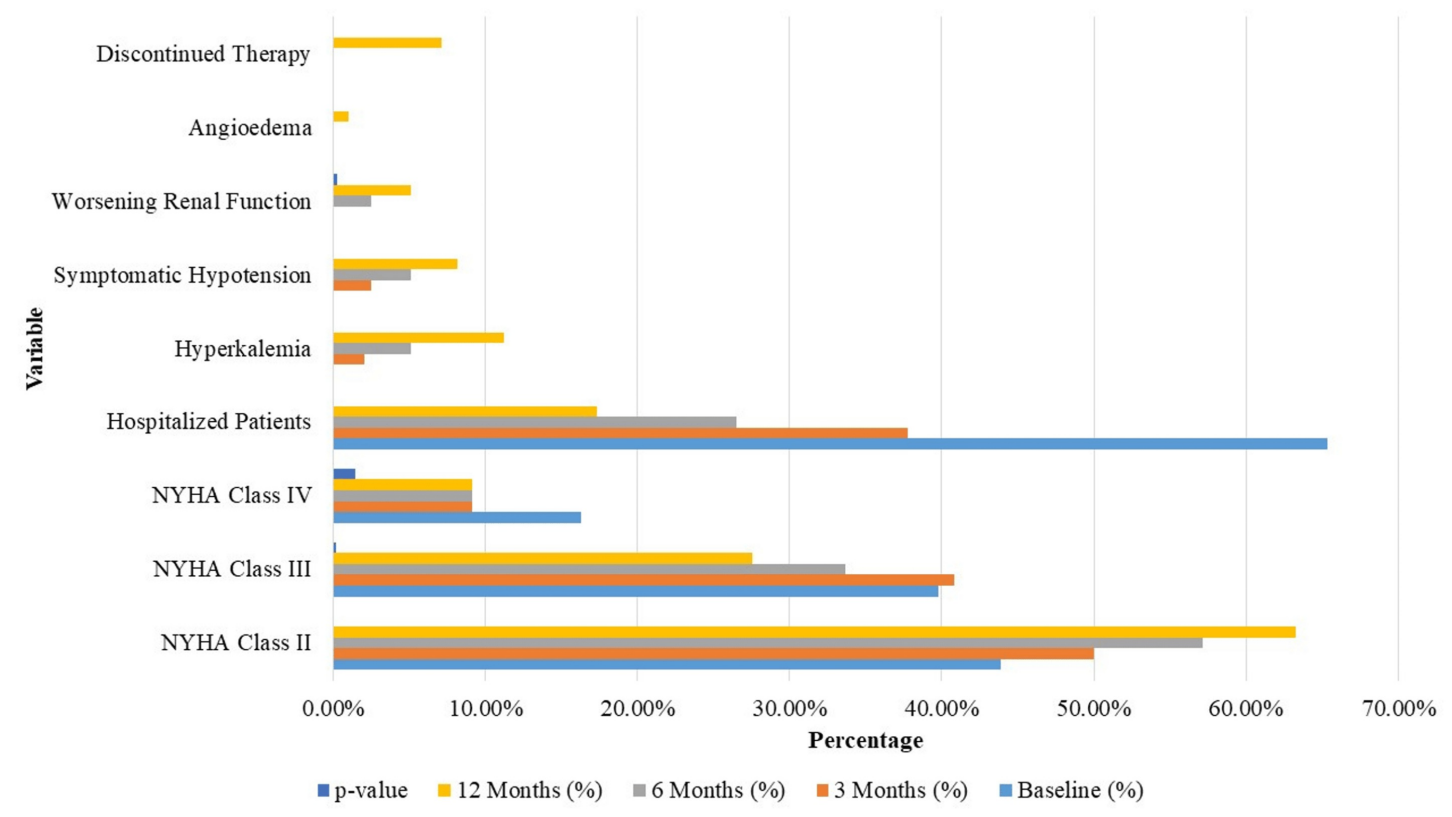
Clinical status and adverse events over 12 months of sacubitril/valsartan therapy. NYHA: New York Heart Association.

Figure 1 shows that over the 12-month treatment period, significant improvements were observed in clinical status and hospitalization rates among HF patients with CKD receiving sacubitril/valsartan. The proportion of patients in NYHA class II increased from 86 (43.88%) at baseline to 124 (63.27%) at 12 months (p < 0.001), while those in class III and IV decreased from 78 (39.80%) to 54 (27.55%) (p = 0.002) and from 32 (16.33%) to 18 (9.18%) (p = 0.015), respectively. Hospitalization rates also significantly dropped, from 128 (65.31%) at baseline to 34 (17.35%) by 12 months (p < 0.001). Regarding adverse events, hyperkalemia increased from 0% to 22 patients (11.22%) (p < 0.001), symptomatic hypotension from 0% to 16 patients (8.16%) (p < 0.001), and worsening renal function (≥20% decrease in eGFR) from 0% to 10 patients (5.10%) (p = 0.003). Furthermore, 14 patients (7.14%) discontinued sacubitril/valsartan due to adverse effects. These findings highlight the treatment’s efficacy in improving HF symptoms and reducing hospitalizations, while also presenting a manageable safety profile.

Clinical status showed considerable improvement over the 12-month follow-up, as shown in Table 5. The proportion of patients in NYHA class II increased from 86 (43.88%) at baseline to 124 (63.27%) at 12 months (p < 0.001). Conversely, NYHA class III declined from 78 patients (39.80%) to 54 (27.55%) (p = 0.002), and class IV from 32 (16.33%) to 18 (9.18%) (p = 0.015). The number of patients hospitalized decreased from 128 (65.31%) at baseline to 34 (17.35%) at 12 months (p < 0.001). Meanwhile, new-onset adverse events included hyperkalemia in 22 patients (11.22%), symptomatic hypotension in 16 (8.16%), and a significant drop in eGFR in 10 (5.10%) patients.

**Table 5:** Changes in clinical status and adverse events in HF patients with CKD over 12 months of sacubitril/valsartan therapy. NYHA: New York Heart Association; HF: heart failure; CKD: chronic kidney disease; eGFR: estimated glomerular filtration rate.

Variable	Baseline (%)	12 months (%)	p-value
NYHA class II	86 (43.88%)	124 (63.27%)	<0.001
NYHA class III	78 (39.80%)	54 (27.55%)	0.002
NYHA class IV	32 (16.33%)	18 (9.18%)	0.015
Hospitalized patients	128 (65.31%)	34 (17.35%)	<0.001
Hyperkalemia	0 (0%)	22 (11.22%)	<0.001
Symptomatic hypotension	0 (0%)	16 (8.16%)	<0.001
Worsening renal function (≥20% ↓ eGFR)	0 (0%)	10 (5.10%)	0.003

## Discussion

This study evaluated the effectiveness of sacubitril/valsartan in HF patients with CKD by examining kidney function, hospitalization rates, and heart performance over a 12-month period. Our results demonstrated significant improvements in eGFR, LVEF, and NYHA classification, along with a marked reduction in hospitalization rates.

Over time, kidney function showed a gradual improvement. eGFR increased from 45.8 ± 12.4 mL/min/1.73 m² at baseline to 46.3 ± 12.0 at three months (+1.09%), 47.1 ± 11.8 at six months (+2.84%), and 48.5 ± 11.5 at 12 months (+5.90%), all with statistical significance (p < 0.05). These results align with earlier studies showing that sacubitril/valsartan slows the decline in kidney function compared to enalapril in HF patients, as evidenced by smaller drops in eGFR over time [12,13]. This supports the conclusion that sacubitril/valsartan may help protect kidney function in individuals with HF and CKD.

The number of hospitalized patients significantly decreased over the study period. At baseline, 128 patients (65.31%) were hospitalized, but this decreased to 74 (37.76%) at three months, 52 (26.53%) at six months, and 34 (17.35%) at 12 months. The total number of hospital admissions also dropped from 210 to 48 over the 12-month period. These findings are consistent with previous studies that demonstrated sacubitril/valsartan's effectiveness in reducing hospitalizations due to HF [14]. Moreover, research by Zhang et al. showed that sacubitril/valsartan was linked to a 20% lower risk of hospitalization compared to ACEIs/ARBs in patients with HFrEF [15]. Our results extend these findings, especially in patients with CKD, a group at higher risk for hospitalization due to kidney dysfunction and fluid retention.

Significant improvements were observed in cardiac function as well. LVEF increased from 32.5 ± 6.7% at baseline to 35.2 ± 6.5% at three months (+8.31%), 38.4 ± 6.3% at six months (+18.15%), and 41.5 ± 6.1% at 12 months (+27.69%), all with statistical significance (p < 0.001). These findings are consistent with previous studies showing sacubitril/valsartan's positive impact on LVEF and reduced HF events compared to enalapril [16]. Additionally, NYHA classification improved, with 63.27% of patients at 12 months in class II (up from 43.88% at baseline), while the proportion of patients in classes III and IV significantly decreased (p < 0.001), further supporting sacubitril/valsartan's role in relieving HF symptoms.

While sacubitril/valsartan was generally well-tolerated, adverse events occurred in some patients. The most common were hyperkalemia (11.22%), symptomatic hypotension (8.16%), and worsening kidney function (≥20% decline in eGFR) (5.10%). These rates were similar to those observed in earlier studies [17]. Despite these risks, the overall benefits of sacubitril/valsartan, i.e., slowing the progression of HF and reducing hospitalizations, outweigh its potential side effects, making it a favorable treatment option for HF patients with CKD.

Strengths and limitations

This study's strengths include its prospective, observational design, which allows for real-world applicability, and its long-term follow-up, providing valuable data on both kidney and heart outcomes in HF patients with CKD. It addresses a significant gap in the literature regarding the safety and efficacy of sacubitril/valsartan in a high-risk population. However, the study has some limitations. The sample size was relatively small (n = 196), and there was no control group. We used convenience sampling, which may introduce selection bias. Nonetheless, the consecutive enrollment of all eligible patients helps mitigate this bias and strengthens the generalizability of our findings. Given the observational nature of the study, direct comparisons to other treatments such as ACEIs and ARBs are not possible. The one-year follow-up may not fully capture the long-term effects of sacubitril/valsartan on kidney and heart function. Future studies with larger sample sizes, randomized controlled designs, and longer follow-up periods are needed to confirm these findings.

## Conclusions

Our study shows that sacubitril/valsartan is an effective therapeutic option for treating HF patients with CKD. It improved both kidney function and heart performance, while also significantly reducing the number of hospitalizations. Most patients tolerated the minor side effects of the treatment well. These results demonstrate that sacubitril/valsartan may improve treatment outcomes in this high-risk population, highlighting its ability to address the complex relationship between HF and CKD. Larger-scale, long-term follow-up studies are needed to validate these findings and refine treatment strategies.
